# Digital Assistive Technology Acceptance and Use by Caregivers of Older Adults With Cognitive Impairment: Qualitative Interview Study

**DOI:** 10.2196/80614

**Published:** 2026-06-19

**Authors:** Xingyue Su, Honglin Chen, Pasi A Karjalainen

**Affiliations:** 1 Department of Social Science, University of Eastern Finland, Yliopistonranta 8, Kuopio, FI-70210, Finland, 358 504420209; 2 Department of Technical Physics, University of Eastern Finland, Kuopio, Finland

**Keywords:** digital assistive technology, cognitive impairment, Technology Acceptance Model, caregiver, older adults

## Abstract

**Background:**

Population aging is associated with a rising number of people living with cognitive impairment (CI), straining traditional caregiving systems. More than 55 million individuals live with CI worldwide, while care delivery faces severe capacity constraints. China’s rapid aging provides a context for examining the acceptance and use of digital assistive technologies (DATs) in CI care. Although DATs offer the potential to address this care gap, systematic evidence on acceptance and use remains limited.

**Objective:**

This study examined caregivers’ acceptance and use of DATs in CI care, applying the Technology Acceptance Model (TAM) framework to identify key facilitators and barriers shaping acceptance and effective technology integration in caregiving environments.

**Methods:**

This qualitative study used semistructured interviews and thematic analysis to examine the use of DATs and identify facilitators and barriers shaping caregivers’ acceptance of DATs. Purposive sampling recruited 15 primary caregivers of people with CI across varying decline stages. The study focused on caregivers of people with CI aged 60 years and older. Caregivers were selected as participants because they serve as the primary decision-makers and technology facilitators in CI care.

**Results:**

In CI care, DATs served 5 primary functional domains. Caregivers identified perceived usefulness and ease of use as primary acceptance facilitators. Factors shaping acceptance emerged across 3 dimensions: technological design factors, user psychological factors, and external environmental factors.

**Conclusions:**

The findings suggest that traditional TAM constructs may benefit from contextual adaptation. The acceptance of DATs is not merely an individual assessment of usefulness or ease of use but also a relational and socially mediated process shaped by emotional dependence, family attitudes, technology anxiety, and support systems. Technology may therefore need to evolve dynamically across different stages of cognitive decline, while human caregivers remain irreplaceable in providing emotional support, personalized judgment, and crisis management. Effective DAT integration requires coordinated efforts among relevant stakeholders.

## Introduction

### Background

China faces severe challenges from population aging, with the population aged 65 years and older exceeding 220 million, accounting for 15.6% of the total population by 2025 [1]. This demographic transformation has generated substantial demands for innovative care solutions, particularly as cases of cognitive impairment (CI) are growing alarmingly from 55 million globally in 2021 to an estimated 139 million by 2050, with China bearing the largest share worldwide [2]. China currently has approximately 9.83 million patients with Alzheimer disease, one of the most prevalent forms of CI, which imposes substantial medical costs and caregiving burdens [3].

### Digital Assistive Technologies in Care for People With CI

In response, digital assistive technologies (DATs) have been increasingly promoted to support the care for people with CI. DATs refer to technology-based tools and systems that are designed to support individuals in maintaining functional ability, independence, and quality of life, particularly in the context of aging and disability [4]. In CI care, these technologies include cognitive training applications, wearable monitoring devices, smart home systems, and other digital solutions that support daily living, safety, and health monitoring [5,6]. While some DATs require active user interaction, others operate passively through ambient sensing and background monitoring, highlighting the need to align technological solutions with users’ cognitive capacities and care environments.

The Chinese government has incorporated CI care into national strategic planning and recent policies promoting smart health and older care models, including wearable devices, service robots, smart antiwandering terminals, medication and care reminder systems, and products for CI assessment and training [7]. Despite growing needs and technological development, a persistent gap remains between the potential of DATs and their effective use in everyday CI care. The usability and effectiveness of DATs are shaped by both technological design and practical deployment [8]. Usability refers to how well a technology enables users to achieve their goals, while effectiveness concerns the accuracy and completeness of that achievement [9]. However, these objective characteristics may not fully explain why some technologies are accepted in practice, while others are not accepted. A device may perform well by objective standards yet still be perceived as difficult to use or of limited value in a specific care context. Focusing solely on objective outcomes, therefore, risks overlooking the subjective factors that determine whether a technology is accepted. Accordingly, this study focuses on caregivers’ perceived usefulness and perceived ease of use, rather than on objective measures of system performance.

### Caregiving in People With CI

CI encompasses a spectrum of conditions affecting memory, attention, language, and executive function, ranging from mild to severe CI, and is associated with progressive loss of independence and increased reliance on caregivers [2]. Caregivers play a central role in mediating the use of DATs for older adults with CI, particularly when individuals experience diminished capacity to evaluate or communicate their preferences. As many older adults with moderate to severe cognitive decline may have difficulty articulating their needs, caregivers serve as key decision-makers, facilitators, and evaluators of technology use in real-world settings. Accordingly, this study examined both formal (trained professionals providing paid care) and informal (nonprofessional individuals, such as family members, relatives, or friends) caregivers to reflect the varied contexts in which DATs are introduced, integrated, and evaluated in CI care.

### Theoretical Frameworks and Related Work

The Technology Acceptance Model (TAM) has been widely used to explain technology acceptance in health care settings [10,11]. TAM proposes that perceived usefulness and perceived ease of use are the primary determinants of users’ behavioral intention to use a technology [12]. It has also been applied to studies of technology uptake in aged care, including mobile health services, wearables, and smart home systems [13].

However, TAM does not fully capture the complexity of CI care settings, where acceptance may also be shaped by personalized training, safety concerns, cultural relevance, disease progression, and digital inequality [14]. A further consideration is that technology use does not necessarily reflect acceptance. Caregivers may continue using a device out of obligation or lack of alternatives, even when they do not perceive it as useful or easy to use. Given that DATs in CI care have not yet been widely embedded in routine practice, understanding the factors shaping acceptance is especially important. This study adopts TAM as its primary theoretical framework, with the interview guide structured around its core constructs. The subsequent thematic analysis was conducted inductively, remaining open to themes beyond TAM’s original scope. As technology continues to evolve, the factors shaping acceptance may also shift. The findings are therefore discussed through the lens of TAM and its extensions to explore whether the specific context of care for people with CI raises considerations not fully addressed by existing models.

### Factors Shaping Technology Acceptance in CI Care

Existing research has identified multiple factors influencing technology acceptance among caregivers and older adults with CI. A systematic review mapping barriers to TAM domains found that most relate to behavioral intentions to use technology, including cost, privacy concerns, stigma, and fear of misuse [15]. Physical and cognitive factors also pose significant challenges, including cognitive decline, reduced physical functioning, and visual, auditory, and language impairments [13]. While prior research has examined caregivers’ acceptance of DATs in the care of people with CI, factors beyond perceived usefulness and perceived ease of use have not been fully examined. This study therefore aims to explore other factors that may shape caregivers’ acceptance of DATs.

### Research Questions

To address these gaps, this study adopts a qualitative approach to explore caregivers’ experiences with DATs in the care of people with CI. Specifically, it examines how caregivers use DATs in everyday care scenarios and identifies the factors influencing their acceptance and sustained use. The study is guided by 2 research questions (RQs):

- RQ1: How are DATs used in CI care settings?
- RQ2: What factors shape caregivers’ acceptance of DATs?

## Methods

### Study Design and Participants

This qualitative study used semistructured interviews to explore caregivers’ experiences with DATs in CI care among older adults aged 60 years and older. Caregivers were selected as the primary participants because they serve as the key decision-makers in technology acceptance and use in this context. As cognitive capacity varies across stages and types of CI, caregivers play a central role in evaluating, implementing, and sustaining the use of DATs in real-world care settings.

Purposive sampling was used with the following inclusion criteria: (1) age ≥18 years, (2) >1 year of experience caring for older people with CI, (3) used at least one DAT, (4) informal or formal caregiver, and (5) communication ability to clearly express care experiences. It should be noted that inclusion criterion (3) restricted participation to caregivers who had already adopted at least one DAT; the experiences and views of caregivers who had not used any DAT were therefore not captured in this study.

Fifteen caregivers were recruited through online communities, offline activities, and institutional recommendations, including 11 informal and 4 formal caregivers. The mean age of the participants was 39.67 (SD 15.0; range 21-78) years (9 women and 6 men), and the mean duration of care was 4.3 (SD 4.0; range 1-16) years.

Data saturation was assessed after each interview by evaluating thematic redundancy, adequacy of data to address the RQs, and the overall coherence of the emerging thematic structure. Initial data saturation indicators emerged by the 10th interview, when core themes around technology acceptance factors began stabilizing. No new themes emerged after the 13th interview, and data collection concluded after the 15th interview. Participant characteristics, caregiving contexts, and types of DATs used are summarized in Table 1.

**Table 1.** Technology use among informal and formal caregivers in cognitive impairment (CI) care^a^.

ID^b^	Gender	Age (y)	Relationship with older adult	Condition of older adult^c^	Duration of care	Technology used	Usage scenario
ICG1	Female	44	Mother-daughter	Severe CI	5 years	Social robot and smart home	Home-based
ICG2	Male	50	Father-son	Mild CI	3 years	GPS shoes and smartwatch	Home-based
ICG3	Male	25	Grandfather-grandson	Mild CI	2 years	Smartwatch	Home-based
ICG4	Female	50	Father-daughter	Severe CI	7 years	Social robot and GPS wristband	Home-based
ICG5	Female	23	Grandfather-granddaughter	Mild CI	1 year	Smart voice assistant	Home-based
ICG6	Male	44	Father-son	Severe CI	3 years	Smartwatch and online mini-program	Home-based
ICG7	Female	46	Father-daughter	Moderate CI	9 years	Social robot and tablet games	Home-based
ICG8	Male	24	Grandmother-grandson	Severe CI with aphasia	2 years	“Xiao Ai” smart assistant and smart mattress	Home-based
ICG9	Female	21	Grandfather-granddaughter	Moderate CI	3 years	Walking aid, smartwatch, and smart home	Home-based
ICG10	Male	78	Spouse	Moderate CI	16 years	Walking aid and smart home	Home-based
ICG11	Female	39	Mother-in-law and daughter-in-law	Severe CI	6 years	Walking aid and emergency alarm system	Home-based
FCG1	Female	44	/^d^	/	1.5 years	“Xiao Ai” smart assistant and smart mattress	Home-based
FCG2	Female	44	/	/	1 year	Smart mattress and senior landline phone	Institutional
FCG3	Male	40	/	/	4 years	Virtual reality (VR) video system	Institutional
FCG4	Female	23	/	/	1 year	Smart wristband and smart mattress	Institutional

a To facilitate the identification of participants’ roles in the analysis, each respondent was assigned a unique identifier. Informal caregivers are labeled as ICG (informal caregiver), and formal caregivers as FCG (formal caregiver). No standardized cognitive assessment was conducted. Detailed descriptions of the digital assistive technology listed in this table are provided in Multimedia Appendix 1.

b The number following each label indicates the individual participant’s ID.

c CI levels reported in this study reflect caregivers’ descriptions informed by prior clinical diagnoses.

d Institutional caregivers who provide care to multiple older adults.

### Data Collection and Analysis

Semistructured interviews lasting 40 to 60 minutes were conducted online and offline. The interview guide covered participants’ background information, DAT use experience, perceived ease of use and usefulness, and factors influencing technology acceptance. TAM informed the interview guide by sensitizing the study to perceived usefulness and perceived ease of use, but it did not determine the full scope of questioning. The semistructured format allowed participants to introduce concerns, experiences, and interpretations beyond the initial TAM-informed prompts. All interviews were recorded and transcribed verbatim, forming approximately 100,000 Chinese words of textual data.

The subsequent thematic analysis was conducted inductively rather than deductively. Codes and themes were generated from participants’ accounts following a systematic 6-step approach: (1) familiarization with data through repeated reading of transcripts and immersion in the dataset; (2) initial code generation to systematically identify and extract key concepts and patterns across the entire dataset; (3) searching for themes by collating codes into potential overarching themes and gathering relevant coded data extracts; (4) reviewing themes through iterative refinement to ensure internal coherence and clear distinctions between themes; (5) naming and defining themes, with later contextualization in relation to the TAM constructs where appropriate; and (6) producing the final analysis report with compelling evidence and analytical narrative [16,17].

The transcribed interviews were read repeatedly by author (XS). Guided by the study aims, relevant data segments were identified and extracted from individual responses, and meaning units were developed with attention to their content and context. These meaning units were interpreted at a semantic level rather than at a latent level. Codes were produced using keywords that captured the essence of the meaning units. The coded meaning units were compared and grouped into categories. After reviewing and refining the categories, they were organized into themes named using short sentences. These themes were finally contextualized and classified according to TAM through a process involving all authors, with authors (HC and PAK) providing final verification and validation. Details of the coding table for data analysis are provided in Multimedia Appendix 2. Research quality was ensured through expert consultation, supervisor discussions, double-checking procedures, diverse participant selection, and regular review of the analysis.

### Ethical Considerations

This nonmedical qualitative study was conducted in accordance with the Finnish National Board on Research Integrity (TENK) guidelines [18]. After the discussion with the Ethical Committee of Research at the University of Eastern Finland, no formal review board number was issued for this study. According to Finnish research ethics principles, interview-based human sciences research should follow the guidelines of TENK, including voluntary participation, informed consent, confidentiality, and protection of personal data. As this study involved qualitative interviews with caregivers rather than medical interventions or direct experimentation with older adults with CI, it would normally be assessed as nonmedical human sciences research under Finnish ethical review principles. Particular attention was nevertheless paid to the sensitive caregiving context, participants’ emotional burden, and anonymization of all interview data. Participation was voluntary, informed consent was obtained prior to data collection, and confidentiality and anonymity were strictly maintained throughout the study. To acknowledge participants’ time investment and valuable contributions, appropriate compensation was provided as an expression of gratitude for their participation. Strict confidentiality protocols were maintained throughout the study, with all personal information and sensitive data anonymized during data processing. Interview recordings were securely stored and deleted on completion of analysis, and all participants are referred to anonymously in research outputs to ensure privacy protection.

## Results

### Overview

This section presents the key findings in responding to the 2 RQs. The first part addresses how DATs are used in CI care settings (RQ1), highlighting 5 core functional domains. The second part examines factors shaping caregivers’ acceptance of these technologies (RQ2), outlining both barriers and facilitators. These themes emerged from the thematic analysis of caregiver interviews.

It is important to note that the functional capabilities of a technology do not automatically translate into perceived benefits for users. A device may be designed to perform a specific function, yet caregivers may not experience it as useful or manageable in their particular care context. This gap between objective functionality and subjective perception is central to why this study focuses on caregivers’ perceived usefulness and ease of use, rather than on what technologies are technically designed to do.

### DAT Applications: From Daily Living to Cognitive Intervention

The following findings address RQ1 by examining how DATs are used in CI care settings. The analysis of caregiver interviews revealed that DATs serve 5 functional domains in CI care, with applications spanning safety support, emotional and social engagement, daily living assistance, health monitoring, and cognitive function support.

#### Safety Support Functions

Safety emerged as the primary concern for caregivers, with DATs showing significant value in this domain. Caregivers reported that smart home devices had an impact on safety and caregiver burden reduction and perceived that these technologies enabled individuals with CI to maintain greater independence.

*My mother’s condition is serious, and these technologies help a lot. The robot alerts me if she doesn’t move for a while. I can also monitor her remotely on my phone, which makes me feel much more at ease.*

[ICG1]

Caregivers described that safety technologies helped reduce the incidence of falls, wandering, and other safety incidents and reported that real-time monitoring enhanced their ability to respond quickly to emergencies.

*My dad fell in the yard. The watch immediately sent an alarm to my phone, and I rushed over. Fortunately, it wasn’t serious. At least I could know it in time…*

[ICG2]

*My father once got lost near the community, but the wristband gave an alarm. We checked the location and found him quickly. The function is very crucial.*

[ICG4]

*We installed smart lights that turn on when they sense movement, and the door locks notify us if Grandpa tries to go out.*

[ICG9]

#### Emotional and Social Support Functions

Social robots are perceived as valuable tools for addressing emotional isolation and social disconnection among individuals with CI. These anthropomorphic devices provided consistent companionship through conversational interactions, entertainment content, and emotional regulation support. Caregivers noted significant reductions in patient agitation, anxiety, and behavioral disturbances when social robots were integrated into care routines.

*At first, I was confused about the robot, but it has helped our family a lot. My mother now chats with it daily, greeting it every morning and even confiding in it. This relieved much of my stress and gave me more time for other tasks*.

[ICG1]

The personalization capabilities of social robots were described as especially beneficial, with devices able to simulate familiar voices or faces, play preferred music, and engage in individualized conversations. Caregivers demonstrated perceived usefulness of DATs in reducing caregiver emotional burden while providing older adults with meaningful social experiences that resembled interactions with family members or friends.

*Before using the robot, my father would wander looking for my deceased mother daily. The robot’s programmed video tasks helped distract him, and now he no longer roams or leaves home. His smiles have even increased from ‘chatting’ with the robot.*

[ICG4]

Caregivers also perceived social robots as supporting emotional stability and social interaction among care recipients while providing themselves with valuable respite and emotional support tools. Caregivers also reported that the continuous availability of these companions helped maintain social connections.

*This robot is very important to my father. When he was depressed, it suggested playing his favorite music. After listening, he began chatting about the past and gradually recovered.*

[ICG7]

#### Daily Living Assistance Functions

Caregivers reported that smart voice assistants reduced the demands of daily care coordination, particularly in managing medication schedules and appointment reminders. The voice-based interface was seen as especially well-suited to care recipients with declining cognitive abilities by participants, as it required minimal operational effort.

*We use an intelligent voice assistant to set daily medication reminders. It’s very convenient—I don’t have to watch over Grandpa all the time. The assistant reminds him when to take which medicine, which is much better than constant supervision.*

[ICG5]

*Sometimes I forget what the medicine is for... the voice assistant tells us its functions, side effects. This saves us the trouble of reading instructions or asking doctors.*

[ICG5]

The medication management capabilities of voice assistants extended beyond simple reminders to include drug information provision, dosage verification, and safety monitoring. According to caregivers, when patients or caregivers had questions about medication purposes, administration methods, or potential side effects, voice assistants could provide immediate, accurate information, reducing the risk of medication errors.

#### Health Monitoring Functions

Caregivers highlighted the reassurance provided by wearable devices and smart sensors through continuous monitoring of vital signs, including heart rate, blood pressure, and body temperature. The ability to receive real-time data and alerts was described as enhancing caregivers’ confidence in managing health risks at a distance.

*I bought a watch for my grandpa to monitor his heart rate. It connects to my phone, so I can know if his heart rate or blood pressure is abnormal.*

[ICG3]

Caregivers also reported health monitoring technologies as tools that supported routine observation and early notification in institutional care settings. These devices were presented as assisting caregivers by generating alerts when unusual conditions were detected, which helped staff become aware of potential problems in a timelier way.

*The smart mattress at the nursing home where I work monitors vital signs, such as when residents get up or remain immobile for long periods. It automatically alerts the responsible nurse in case of abnormalities.*

[FCG3]

#### Cognitive Function Support

Cognitive training applications delivered through various digital platforms, including WeChat mini-programs (lightweight applications embedded within WeChat, a widely used instant messaging and social media platform in China), tablet-based cognitive games, and mobile-based apps, were perceived as showing promise in maintaining cognitive engagement among care recipients.

WeChat mini-programs allow users to access services, including cognitive games without installing separate software, offering convenient cognitive training, including memory exercises, attention training, and logic tasks.

*My father plays a memory card matching game on WeChat daily. Though he sometimes forgets details, he enjoys it greatly. The group also has small games like arithmetic and riddles, which keep him engaged and prevent excessive daydreaming or sleep.*

[ICG6]

Tablet-based cognitive games provided more comprehensive cognitive training through touchscreen interfaces and intuitive game mechanics. These applications were reported to address multiple cognitive domains, including memory, attention, problem-solving, and reasoning skills through engaging, gamified activities such as puzzles, spot-the-difference games, and simple mathematical problems by caregivers.

*I downloaded a tablet game with puzzles and spot-the-difference for my dad. He plays it daily, which helps improve his reaction speed and concentration.*

[ICG7]

Caregivers described the gamified format as helpful for sustaining care recipients’ engagement and motivation. In their accounts, game elements such as challenges and progression systems made cognitive exercises feel more enjoyable and supported more regular participation.

*Grandpa played a tablet game where he had to identify potential dangers like kitchen knives or falling objects. This helped enhance his risk judgment.*

[ICG3]

According to caregivers, language-focused games appeared to support verbal communication and delay language decline, although these observations reflect caregiver perceptions rather than clinical assessments.

*These games include language exercises such as word puzzles that delay language decline and improve vocabulary.*

[ICG7]

### Factors Influencing Technology Acceptance in CI Care

The following findings address RQ2 by examining the factors that shape caregivers’ acceptance of DATs in CI care. These factors are organized into 3 categories: technology design, user psychology, and external environment, each of which encompasses both facilitators and barriers to acceptance.

#### Technology Design Factors

##### Design Usability and Operational Challenges

Participants identified operational complexity and user interface design as significant barriers to technology acceptance among individuals with CI and their caregivers. They reported that complex operational procedures and nonintuitive interfaces created difficulties for older adults with declining cognitive abilities, which could reduce willingness to use these technologies.

*Smart home devices have many functions, but the operation is too complicated. My parents often forget which button to press and sometimes give up. Some functions expected to be automatic aren’t used, so I must operate them manually, which takes more time.*

[ICG1]

*My dad struggles with the tablet. There are too many options, and he gets confused easily. He often presses the wrong buttons and ends up on unfamiliar pages with ads. He can only use it with my help.*

[ICG2]

Participants further described a gap between their initial expectations and their actual experience of the technologies. Caregivers reported that they had expected these tools to reduce care burden and support care recipient autonomy, but technical limitations and inconsistent performance often limited these perceived benefits.

*The smart assistant doesn’t understand grandma’s dialect well; she only speaks Teochew, and her Mandarin isn’t standard.*

[ICG8]

*The positioning shoes sometimes fail to give timely alarms, which worries me.*

[ICG2]

*My grandfather often can’t hear the voice assistant’s medicine reminders clearly and sometimes just ignores repeated instructions.*

[ICG3]

*I thought the smartwatch would reduce my caregiving burden by reminding medicine and monitoring health, but unstable networks and inaccurate data made me spend more time managing it.*

[ICG6]

*I hoped the social robot could ease my dad’s emotional issues so I wouldn’t have to be with him constantly. But it often misunderstands him, and my daughter or I must stay nearby to help.*

[ICG4]

*The sensor light helps at night when my spouse gets up, but the camera isn’t sensitive enough and sometimes misses parts of the room, which worries me.*

[ICG10]

Taken together, participants’ accounts suggested that perceived usefulness in the care of people with CI was shaped not only by a technology’s intended functions but also by the extent to which it performed reliably and aligned with caregivers’ expectations in everyday care practice.

##### Personalization and User-Specific Needs

Participants identified insufficient personalization and limited inclusive design as significant constraints on the perceived usefulness of DATs in the CI care. They reported that many smart devices followed a “one-size-fits-all” design and did not adequately respond to individual care needs, which could reduce willingness for sustained use.

*Our robot can only customize one spouse image for my dad, which isn’t enough. He used to have many friends. If we could add images of old friends or comrades, he’d be happier. We’d pay for better customization, but current options are very limited.*

[ICG4]

*I bought my dad a big tablet, but it has too many functions he can’t use. He can’t remember complex steps. Many functions are unnecessary for him. The design doesn’t fit his needs.*

[ICG7]

Although some devices provided simple customization options, these typically failed to fully meet the actual needs of care recipients and caregivers. Participants also expressed a need for more flexible customization across different stages of CI and noted that the absence of stage-appropriate functions limited the usefulness of existing devices in everyday care.

### User Psychological Factors

#### Technology Anxiety and Psychological Adaptation

In the interviews, technology anxiety was presented as an important barrier to technology acceptance among both individuals with CI and their caregivers. Caregivers reported that unfamiliar technologies often gave rise to confusion and apprehension, which could reduce willingness to use these devices.

Caregivers’ accounts suggested that technology anxiety became stronger when devices required abilities that people with CI could no longer easily manage. They described complex technology as increasing psychological burden and sometimes leading care recipients to avoid or resist use. Repeated operational difficulties were also reported as weakening users’ confidence and heightening uncertainty about their own ability to use the device. Caregivers further noted that they experienced similar pressure when device setup was complicated or when they had to repeatedly solve technical problems and explain unfamiliar functions during care.

*Every time my grandfather tries to use the smartwatch, he gets nervous. He fears pressing wrong buttons or accidental payments. Even with repeated teaching, when alone, he dares not operate the device and prefers to give up.*

[ICG9]

#### Caregiver Technology Burden and Support

Although DATs were reported to reduce caregiver burden and improve care efficiency, caregivers reported that learning new technologies alongside daily care work increased their workload, particularly when they also needed to manage maintenance and provide repeated support during use. Limited technical support was described as a further challenge. Several participants noted that device malfunctions often had to be handled independently, and some formal caregivers reported that they had received little training and mainly relied on trial and error.

*My father can’t use mini-programs independently. Whenever issues arise, I have to help him reset or troubleshoot. I’ve taught him repeatedly, but he can’t remember, so I’m always the one managing it.*

[ICG6]

*My dad enjoys interacting with the robot, but it often fails to recognize his speech or responds incorrectly. I must stay with him to correct errors. When no one helps, he becomes anxious and frustrated, sometimes telling the robot to shut up. I worry he’ll lose willingness to try.*

[ICG4]

*When equipment malfunctions, we resort to online guides or customer service, but problems often remain unresolved. Instead of reducing workload, these devices increase our burden.*

[FCG2]

*The smart mattresses at our nursing home need training, but it was brief and insufficient. When malfunctions occur, we rely on customer support or technicians, which is time-consuming. Sometimes the alerts misidentify patients, and fixing these issues is complex.*

[FCG4]

### External Environmental Factors

#### Family Attitudes and Cultural Values

Caregivers’ accounts indicated that family member attitudes and support were important to the acceptance of DATs. They reported that encouragement or resistance from family members, as well as the presence or absence of emotional and practical support, could shape care recipients’ acceptance of these technologies in everyday care.

Across interviews, some caregivers described family involvement as facilitating technology use. Support with selecting, purchasing, installing, and explaining devices was presented as helping reduce hesitation and making technologies easier to introduce into daily care.

*I was hesitant at first because these high-tech products are expensive and unfamiliar. But my daughter was very supportive, helping with installation and teaching my dad step-by-step. Now, although my dad learns slowly, he can basically use the devices, and I feel more secure knowing my daughter is there to help*.

[ICG7]

*My daughter suggested installing smart home systems and helped me choose and set up lights and alarms. I was initially afraid of improper operation, but with her help, I gradually found the devices easy to use. Sometimes, they really save us a lot of trouble*.

[ICG10]

Interview accounts also showed that family attitudes could constrain the introduction of DATs. Some caregivers reported that objections from relatives, concerns about cost, or discomfort with the idea of technology-mediated care limited their acceptance of such devices. In these accounts, hesitation was related not only to practical issues but also to concerns about whether technology-based care might be seen as inadequate, impersonal, or inconsistent with family responsibility.

*I wanted to install a monitoring system to know if grandma is safe when I go out, but my parents opposed it. Grandma said, “What’s the point? Treating me like a show?” She often gets angry and resents the nanny. Since grandma disliked it, I didn’t argue and didn’t buy the equipment*.

[ICG8]

*I wanted to buy my mother-in-law a talking robot, but my husband said it’s too expensive and unnecessary*...

[ICG11]

*My mother feels that using robots makes her seem irresponsible or disrespectful to the elderly, who need human companionship more. Although these technologies might reduce burden, she feels uneasy*.

[ICG9]

*People here think elderly care should be done personally. When I mentioned smart devices to peers, many thought I was being lazy or irresponsible. I felt conflicted because the intention was to reduce burden, but it was seen negatively.*

[ICG4]

Taken together, these accounts suggest that acceptance of DATs was shaped not only by practical considerations but also by family norms and cultural expectations surrounding care. For some caregivers, technology was viewed as potentially helpful, while for others, it raised concerns about responsibility, appropriateness, and the place of human care in family life.

#### Affordability and Sustained Use

Economic conditions were an important consideration in caregivers’ acceptance and use of DATs. Several caregivers raised concerns about the cost of devices, particularly in families with limited financial resources where purchase, maintenance, and continued use could place a substantial burden on the household.

*Some technologies like health monitors are excellent, but our family can’t afford the high cost, especially for long-term use and maintenance*.

[ICG2]

*If prices were reasonable, with public resources or subsidies, I would consider buying more useful devices. Often the technology is good, but we can’t afford it*.

[ICG6]

*I wanted to buy my mother-in-law a talking robot, but my husband thought it was too expensive and unnecessary. We rely on my income alone, so it’s not affordable*.

[ICG11]

Across interviews, caregivers indicated that they would be more willing to try or continue using DATs if costs were lower or if financial support, such as subsidies, were available. Some participants acknowledged the potential value of these technologies but described cost as a practical barrier to uptake and continued use. Ongoing expenses, including service fees and maintenance costs, were also mentioned as limiting sustained use in everyday care.

## Discussion

### Principal Findings

This study explored how DATs are used in care for people with CI and what factors shape caregivers’ acceptance. The discussion first relates 4 identified themes to TAM-related models, including family attitudes, social support, technology anxiety, and emotional dependence. It then considers stage-specific care needs, the essential role of human caregivers, and coordinated support for DAT integration.

### Technology Acceptance Beyond Established TAM Constructs

#### Overview

The findings of this study are broadly consistent with TAM’s core constructs. Caregivers identified perceived usefulness and perceived ease of use still as central factors shaping their acceptance of DATs. However, the findings differentiate this study from conventional technology acceptance studies by showing how social, emotional, and care-contextual dimensions shape acceptance alongside perceived usefulness and perceived ease of use. Beyond TAM’s core constructs, participants described 4 themes shaping their acceptance: family attitudes, social support, technology anxiety, and emotional dependence. Examined through the lens of TAM, TAM3, and Unified Theory of Acceptance and Use of Technology (UTAUT), the identified factors showed different degrees of correspondence with established constructs, although some factors point to dimensions of technology acceptance that existing models have not fully addressed in the care context for people with CI. These observations are offered tentatively, as they are grounded in a small qualitative sample and warrant further empirical investigation.

#### Family Attitudes

Family attitudes toward technology shaped DAT acceptance in care for people with CI by influencing both attitudes toward technology and the practical conditions for continued use. Sustained technology use is often less a matter of older adults’ individual acceptance than of family members’ decisions and ongoing involvement, particularly in China, where older adults often rely on family support. Family caregivers typically take responsibility for selecting, setting up, and maintaining technologies, as well as providing day-to-day guidance during use. Prior research on technology socialization highlights that adult children and other family members frequently act as key facilitators by embedding technologies into everyday family practices [19]. Evidence from China further shows that intergenerational support enhances perceived ease of use, perceived usefulness, and trust in digital health services [20]. These findings correspond in part to UTAUT’s social influence construct because relatives’ expectations shaped attitudes toward DATs [21]. It also relates to facilitating conditions because family members often provide practical support for use.

#### Social Support

Social support, particularly from social workers and community programs, enhances technology acceptance by providing training and psychological support during initial acceptance phases. The existence of social support enables some caregivers to receive timely assistance when they encounter difficulties, enhancing their confidence and persistence in the use of technology [20]. This corresponds to UTAUT’s facilitating conditions construct, which highlights the importance of support structures for technology use [21] because community programs, social workers, and service providers shaped whether caregivers had the training, troubleshooting support, and reassurance needed to continue using DATs. In this study, such support was not limited to formal organizational or technical arrangements, but also included community-based and interpersonal forms of assistance.

#### Technology Anxiety

Technology anxiety, identified as a barrier to acceptance among both people with CI and caregivers, corresponds closely to the computer anxiety construct in TAM3, where anxiety is understood as shaping perceived ease of use [22]. In this study, this was reflected in caregivers’ accounts that unfamiliar or cognitively demanding devices often created apprehension and reduced willingness to engage. Among people with CI, such anxiety was especially strong when repeated operational difficulties occurred, while among caregivers, it was linked to complex setup requirements and responsibility for troubleshooting.

#### Emotional Dependence

Emotional dependence was the theme least readily aligned with existing TAM or UTAUT constructs. Prior research on human-robot interaction has identified emotional connection as a factor shaping older adults’ engagement with assistive technologies, alongside more functional considerations such as perceived usefulness and ease of use [23]. From a relational care and sociotechnical perspective, this finding suggests that DATs enter existing care relationships rather than being added simply as neutral tools. Their acceptance depended partly on whether caregivers and care recipients perceived them as fitting into routines of companionship, reassurance, and emotional presence. Some caregivers remained hesitant about technology use when they felt that devices could not provide warmth or emotional presence. Others reported that when older adults developed emotional attachment to a device, they were sometimes more willing to engage with it. This theme therefore extends beyond traditional TAM constructs in a narrow functional sense. Devices became more acceptable when they gained emotional meaning within care routines but were resisted when perceived as unable to provide human warmth or relational presence.

### Technology Needs Across Stages of Cognitive Decline

#### Caregivers Described Technology Needs as Evolving Alongside Cognitive Decline

Caregivers described care priorities and technology use as changing across stages of cognitive decline, ranging from cognitive support and memory assistance in mild stages to safety monitoring and passive sensing in later stages. Their accounts suggested 3 broad patterns. In mild stages, caregivers tended to prioritize independence and described using memory aids and cognitive training tools to support everyday functioning. In moderate stages, greater emphasis was placed on safety monitoring and personalized interaction, with social robots and monitoring systems described as more relevant in responding to behavioral change. In severe stages, caregivers referred more often to comprehensive monitoring and nonverbal forms of interaction in the context of high care dependency. These patterns appeared to vary across individuals and care settings. Taken together, the findings suggest that technologies for the care of people with CI may need to be flexible enough to respond to changing care needs across stages of decline.

#### Human Caregivers and Technology in Collaborative Care

Participants’ accounts suggested that human caregivers remained essential in 3 aspects of care: emotional support, personalized judgment, and response to complex situations. Technology was not perceived as fully capable of meeting these needs, particularly when care involved unexpected circumstances. These accounts suggest that participants viewed DATs mainly as supportive tools, while human caregivers continued to be seen as central to care.

*My mother feels that using robots makes her seem irresponsible or disrespectful to the elderly, who need human companionship more. Although these technologies might reduce burden, she feels uneasy*.

[ICG9]

*The smart mattress means I don’t have to stay by the patient’s side all the time, but when something more complex happens, you still need a person there to handle it*.

[FCG2]

#### Implications for Coordinated Technology Integration in Care for People With CI

The findings suggest that effective integration of DATs in the care of people with CI may depend on coordinated support across multiple stakeholder groups [24]. Previous research has highlighted the role of clinicians in evaluating and responsibly introducing DATs in this context [25]. In this study, participants’ accounts pointed to 3 areas of practical relevance. For technology developers, the findings highlight the importance of age-friendly interfaces and functions that can respond to changing care needs across stages of cognitive decline. For social workers, the findings suggest a role in supporting technology use through guidance, training, and emotional support during implementation. For policymakers, the findings point to the need to improve affordability and reduce structural barriers to access, including through financial support and appropriate regulation. Taken together, these implications suggest that DAT integration requires coordinated support across technology design, caregiver training, implementation support, and policy measures addressing affordability and access.

### Limitations

This study acknowledges several limitations. First, the sample was relatively small, comprising 15 caregivers primarily from family care settings in China, which limits representativeness across diverse geographical and cultural contexts. More detailed sociodemographic characteristics, such as education level, socioeconomic background, and digital literacy, were not systematically examined, and these factors may influence technology acceptance. As inclusion criteria (3) required prior DAT use, the sample reflects the experiences of adopters only, and the views of caregivers who had not used any DAT are not captured. The diversity of the sample in terms of caregiver type, age, and technologies used also means that findings cannot be attributed to any specific caregiver group or device category.

The qualitative methodology, while providing rich insights into caregiver experiences, inherently carries subjective interpretation risks and lacks quantitative validation. Additionally, the study focuses on currently available technologies rather than emerging innovations such as AI-powered robots or VR/AR applications, which remain limited in practical implementation due to cost and accessibility barriers [24].

A further limitation concerns the source of the data, as this study captures caregiver perspectives rather than direct experiences of care recipients, who may face communication challenges that complicate assessment of their actual technology preferences. In addition, CI stages reported in Table 1 were based on caregivers’ descriptions informed by prior clinical diagnoses, and no standardized cognitive assessments were conducted by this research. Future research should include larger, more diverse samples; use mixed method approaches, longitudinal designs to assess long-term technology adaptation, and develop innovative methods to capture the authentic experiences of care recipients themselves.

### Conclusions

This study examined how DATs are used in care for people with CI and what factors shape caregivers’ acceptance of these technologies. Caregivers described DATs as being used across 5 functional domains: safety support, emotional and social engagement, daily living assistance, health monitoring, and cognitive function support. Their accounts also showed that acceptance was shaped not only by perceived usefulness and perceived ease of use but also by family attitudes, social support, technology anxiety, and emotional dependence. Technologies were described as more acceptable when they were reliable, easy to manage, and responsive to stage-specific needs. They were less acceptable when they increased caregiver burden, failed to match everyday care demands, or conflicted with family expectations and care values. DATs may be used in care, but acceptance still depends on perceived usefulness, ease of use, relational care, family attitudes, and support conditions. Overall, the findings suggest that DAT integration depends on relational, cultural, and social contexts rather than technical functionality alone. Across interviews, DATs were generally viewed as supportive tools within care, while human caregivers remained essential for emotional support, individualized judgment, and response to complex situations.

These findings have 3 practical implications. First, DAT design should prioritize simplicity, personalization, and flexibility across different stages of CI. Second, implementation should be accomplished by accessible training and sustained support for caregivers. Third, wider uptake is likely to depend on enabling family, community, and policy conditions. In summary, improving DAT integration in care for people with CI requires attention not only to what technologies are designed to achieve but also to how caregivers and recipients experience, assess, and sustain their use in everyday care. Effective DAT integration must be grounded in caregivers’ perceptions and in the broader social and cultural contexts of technologies. Future research should further examine these dynamics in larger and more diverse samples.

## Supplementary material

10.2196/80614 Multimedia Appendix 1 Description of digital assistive technologies (DATs).

10.2196/80614 Multimedia Appendix 2 Coding table.

## Abbreviations

CI: cognitive impairment
DAT: digital assistive technology
RQ: research question
TAM: Technology Acceptance Model
UTAUT: Unified Theory of Acceptance and Use of Technology

## References

[1] National Bureau of Statistics of China (2025). China Statistical Yearbook 2025.

[2] Li J, Li J (2025). Trends, inequalities, and cross-location similarities in global dementia burden and attributable risk factors across 204 countries and territories: a systematic analysis for the Global Burden of Disease Study 2021. Int J Surg.

[3] Ren R, Qi J, Lin S (2022). The China Alzheimer Report 2022. Gen Psychiatr.

[4] (2024). Assistive technology. World Health Organization.

[5] Thordardottir B, Malmgren Fänge A, Lethin C, Rodriguez Gatta D, Chiatti C (2019). Acceptance and use of innovative assistive technologies among people with cognitive impairment and their caregivers: a systematic review. Biomed Res Int.

[6] Zang GY, Rao K, Wu J, He Y, Tang Y, Shi L (2025). Digital therapeutics for cognitive impairment: exploring innovations, challenges, and future prospects. J Med Internet Res.

[7] General Office of the State Council (2024). Opinions of the General Office of the State Council on developing the silver economy and promoting the welfare of the elderly. Central People’s Government Portal.

[8] Meiland F, Innes A, Mountain G (2017). Technologies to support community-dwelling persons with dementia: a position paper on issues regarding development, usability, effectiveness and cost-effectiveness, deployment, and ethics. JMIR Rehabil Assist Technol.

[9] (2019). Ergonomics of human-system interaction — part 210: human-centred design for interactive systems. International Organization for Standardization.

[10] Ammenwerth E (2019). Technology Acceptance Models in health informatics: TAM and UTAUT. Stud Health Technol Inform.

[11] Rahimi B, Nadri H, Lotfnezhad Afshar H, Timpka T (2018). A systematic review of the Technology Acceptance Model in health informatics. Appl Clin Inform.

[12] Davis FD (1989). Perceived usefulness, perceived ease of use, and user acceptance of information technology. MIS Q.

[13] Zhou C, Ai Y, Wang S (2024). Barriers and facilitators to participation in electronic health interventions in older adults with cognitive impairment: an umbrella review. BMC Geriatr.

[14] Boyle LD, Husebo BS, Vislapuu M (2022). Promotors and barriers to the implementation and adoption of assistive technology and telecare for people with dementia and their caregivers: a systematic review of the literature. BMC Health Serv Res.

[15] Malden S, McGill K, Guthrie B (2025). Patient and carer-related facilitators and barriers to the adoption of assistive technologies for the care of older adults: systematic review. JMIR Aging.

[16] Braun V, Clarke V (2014). What can “thematic analysis” offer health and wellbeing researchers?. Int J Qual Stud Health Well-being.

[17] Braun V, Clarke V (2022). Thematic analysis: a practical guide. bpsqmip.

[18] The Ethical Principles of Research with Human Participants and Ethical Review in the Human Sciences in Finland.

[19] Tang Z, Shah M, Jamal A (2024). Exploring the process of technology socialization (TS) in the family: ICT adoption for middle-aged parents with the influence of adult children. Inf Syst Front.

[20] Jin H, Qu Y (2025). Association between intergenerational support, technology perception and trust, and intention to seek medical care on the internet among Chinese older adults: cross-sectional questionnaire study. J Med Internet Res.

[21] Venkatesh V, Morris MG, Davis GB, Davis FD (2003). User acceptance of information technology: toward a unified view1. MIS Q.

[22] Venkatesh V, Bala H (2008). Technology Acceptance Model 3 and a research agenda on interventions. Decision Sciences.

[23] Sirizi D, Sabet M, Yahyaeian AA, Bacsu JDR, Smith ML, Rahemi Z (2025). Evaluating human-robot interactions to support healthy aging-in-place. J Appl Gerontol.

[24] Watanabe K, Miwa H, Wakui T (2023). Technology integration in care service systems: the required actions of technology developers. IEEE Eng Manag Rev.

[25] Ienca M, Fabrice J, Elger B (2017). Intelligent assistive technology for Alzheimer’s disease and other dementias: a systematic review. J Alzheimers Dis.

