# Supplementary material for: Digital Assistive Technology Acceptance and Use by Caregivers of Older Adults With Cognitive Impairment: Qualitative Interview Study
Source: JMIR Hum Factors. 2026 Jun 19;13:e80614. doi: 10.2196/80614 (PMC13282040; doi:10.2196/80614)
Supplement: Multimedia Appendix 1 [file humanfactors-v13-e80614-s001.docx]

Appendix 1. Description of Digital Assistive Technologies Reported in Table 1

Table 1 Description of Digital Assistive Technologies

| Technology category | Core functions | Care scenario |
| --- | --- | --- |
| Smartwatch | GPS tracking; fall detection; heart rate monitoring; emergency alerts | Home-based cognitive care; prevention of wandering and falls; remote caregiver monitoring |
| GPS shoes / GPS wristband | Real-time location tracking; geofencing; caregiver alerts | Home and community settings for individuals at risk of wandering |
| Social robot | Voice interaction; emotional companionship; reminders; multimedia playback | Home-based cognitive care to reduce loneliness, agitation, and caregiver stress |
| Smart voice assistant | Voice-controlled reminders (Xiaoai: <https://xiaoai.mi.com/?from=aihome.run>); daily task assistance; information queries | Daily routine support for individuals with mild–moderate cognitive impairment |
| Smart home system | Motion-sensor lighting; door monitoring; safety alerts; remote control | Home safety enhancement, especially night-time fall prevention |
| Smart mattress | Sleep monitoring; bed-exit detection; abnormal activity alerts | Institutional or semi-institutional care settings |
| Tablet-based cognitive games | Memory, attention, and problem-solving training; touchscreen interaction | Cognitive training and engagement for mild–moderate cognitive impairment |
| Mobile applications / WeChat mini-programs | Cognitive games; reminders; daily task support; no separate installation required (<https://mp.weixin.qq.com/cgi-bin/wx>) | Routine cognitive stimulation in home-based care, especially in China |
| VR video system | Immersive visual content;reminiscence support | Institutional care for emotional engagement |
