# Supplementary material for: Digital Assistive Technology Acceptance and Use by Caregivers of Older Adults With Cognitive Impairment: Qualitative Interview Study
Source: JMIR Hum Factors. 2026 Jun 19;13:e80614. doi: 10.2196/80614 (PMC13282040; doi:10.2196/80614)
Supplement: Multimedia Appendix 2 [file humanfactors-v13-e80614-s002.docx]

**Appendix 2. Coding Table**

*English version translated and standardized from the Chinese coding table.*

| **Theme** | **Main Category** | **Subcategory** | **Concepts Derived From Textual Material** |
| --- | --- | --- | --- |
| Practical Applications and Experiences of Digital Assistive Technologies | Functional Applications of Digital Assistive Technologies | Safety Support Functions | Smart sensors monitor older adults' movement trajectories and detect possible falls in real time. |
|  |  |  | Smart lighting systems sense older adults' behavior and adjust environmental lighting. |
|  |  |  | Automatic alerts are sent to caregivers in emergencies to support timely responses. |
|  |  |  | Wearable devices and remote data analysis platforms collect key vital signs in real time. |
|  |  | Health Monitoring Functions | Health data are transmitted promptly to medical teams to support rapid intervention and health management. |
|  |  |  | Automated reminders help older adults take medication on time and provide medication-related information. |
|  |  | Emotional and Social Support Functions | Voice assistants and social robots interact with older adults through daily conversations and music playback. |
|  |  |  | Virtual companion devices perceive older adults' emotional states and provide personalized emotional support. |
|  |  |  | Technologies support psychological well-being and help relieve loneliness and anxiety. |
|  |  | Daily Living Assistance Functions | Daily task management provides reminders and helps regularize everyday routines. |
|  |  |  | Smart mobility aids or wheelchairs help older adults move safely at home and outdoors. |
|  |  | Cognitive Function Support | Cognitive training platforms provide tools targeting memory, logical reasoning, and spatial perception. |
|  |  |  | Interactive and engaging training games are suitable for older adults with mild cognitive impairment. |
|  | Factors Influencing Technology Acceptance | Operational Complexity and User Interface Design | Devices are difficult to operate and cannot be used independently. |
|  |  |  | User interfaces are cumbersome and contain too many functions. |
|  |  |  | Technologies are not well adapted to the needs of older adults with cognitive impairment. |
|  |  | Technology Anxiety and User Adaptation | Limited understanding of new technologies creates barriers to use. |
|  |  |  | The operation process requires a long adaptation period, especially for older users. |
|  |  |  | Developing stable usage habits takes time, and older adults' willingness to continue using the technology may be low. |
|  |  | Caregiver Technology Burden and Support Needs | Assistive technology devices are difficult to maintain. |
|  |  |  | Caregivers receive insufficient technical support and training. |
|  |  |  | Assistive technologies may encounter technical problems during use. |
|  |  | Insufficient Personalization and Inclusive Design | Customization functions are insufficient and personalized design is lacking. |
|  |  |  | Technologies show limited inclusiveness and cannot accommodate older adults with different cognitive conditions. |
|  |  | Perceived Usefulness and Function-Need Fit | There is a gap between perceived functions and actual user experience. |
|  |  |  | Health monitoring devices may lack sufficient sensitivity. |
|  |  | Gap Between Expected Improvement and Actual Experience | The accuracy of data provided by devices needs improvement. |
|  |  |  | Network latency may lead to inaccurate data. |
|  |  |  | The expected improvements brought by technology are not fully realized. |
|  |  | Economic Conditions and Technology Accessibility | The high cost of technology devices makes them difficult to afford. |
|  |  |  | Government subsidy policies for relevant technologies are insufficient. |
|  |  |  | Promotion among economically disadvantaged groups is limited, and high prices make purchase difficult. |
|  |  | Family Structure and Technology Acceptance | Family members' support and participation directly influence technology acceptance. |
|  |  |  | Traditional caregiving expectations create concerns that technology cannot replace interpersonal care. |
|  |  | Influence of Cultural Background on Technology Acceptance | Families often feel responsible for personally caring for and accompanying older adults. |
|  |  |  | Traditional social norms may reject technology-mediated care. |
|  |  | Role of Social Support Systems | Social workers provide technology-use training, psychological support, and resource linkage for older adults and caregivers. |
|  |  |  | Communities organize regular technology training sessions, lectures, and exchange activities. |
|  |  |  | Government support promotes the broader application of digital technologies through policies and financial subsidies. |
